# Enhanced chemotherapeutic efficacy of the low-dose doxorubicin in breast cancer via nanoparticle delivery system crosslinked hyaluronic acid

**DOI:** 10.1080/10717544.2018.1507057

**Published:** 2019-01-28

**Authors:** Qin Wang, Yinan Zhong, Wenting Liu, Zemin Wang, Liqin Gu, Xuejiao Li, Jiqing Zheng, Huan Du, Zhiyuan Zhong, Fang Xie

**Affiliations:** a Department of Pathology, Institutes of Biology and Medical Sciences, Soochow University Medical College, Soochow University, SuzhouP. R. China;; b Department of Immunology, Institutes of Biology and Medical Sciences, Soochow University Medical College, Soochow University, Suzhou, P. R. China;; c Biomedical Polymers Laboratory, Jiangsu Key Laboratory of Advanced Functional Polymer Design and Application, College of Chemistry, Chemical Engineering and Materials Science, Soochow University, SuzhouP. R. China;; d Department of Pathology, The Frist Affiliated Hospital of Soochow University, Suzhou, P.R. China;; e Investigative Toxicology and Pathology Laboratory, School of Public Health, Indiana University, Bloomington, IN, USA;; f Department of Pathology, Taicang Traditional Medicine Hospital of Jiangsu Province, Taicang, P.R. China

**Keywords:** Chemotherapy, doxorubicin, nanoparticle, hyaluronan, cancer stem cell, breast cancer

## Abstract

Despite the development of treatment options in breast cancer, many patients die of recurrence and metastasis. Owing to enhanced permeability and retention in solid tumor tissue, nanoparticle (NP) delivery systems have been emerged as novel strategy in cancer chemotherapy. As extracellular matrix, glycosaminoglycan hyaluronan (HA) could bind its surface receptor adhesion molecule CD44 which is strongly expressed on breast cancer. We have previously reported a doxorubicin (DOX)-loaded HA-Lys-LA X-NPs (X-NP-DOX) NP delivery system for breast cancer treatment. In this study, we further investigated the antitumor effect of X-NP-DOX NP delivery system using low-dose DOX in both *in vitro* and *in vivo* systems. We demonstrated that low-dose X-NP-DOX possessed the ability for inhibiting MCF-7 breast cancer cell growth, invasion, and migration, and inducing apoptosis *in vitro*. In *in vivo* experiments, injection of low-dose X-NP-DOX into tumor-bearing mouse resulted in significant reduction of tumor size. Terminal deoxynucleotidyl transferase-mediated dUTP nick end labeling staining further revealed that low-dose X-NP-DOX induced higher percentage of apoptotic cells compared with free DOX or saline. Furthermore, our study demonstrated that low-dose X-NP-DOX inhibited Notch1 and Ras/MAPK pathways, decreased cancer stem cell population, and reduced tumorigenesis compared to free DOX in both *in vitro* and *in vivo* settings. Owing to its enhanced efficacy and higher targetability compared to free DOX, low-dose DOX delivered by NP system may be a promising novel strategy for breast cancer treatment.

## Introduction

Recently, nanoparticles (NPs) are regarded as a novel solution to improve therapeutic efficacy and reduce systemic toxicity in antitumor treatment owing to their smaller size, shorter circulation time, and larger modified surface area (Chen et al., 2014; Long et al., 2014; Morton et al., 2014; Zhao et al., 2014). Different from traditional drug-delivery system, the NPs with modified surface can bind to receptors expressed at the target site, and can be internalized into cancer cells through its pharmacological action (Rapoport et al., 2007; Long et al., 2014).

Most malignant solid tumors have unique blood vessels with the enhanced permeability and retention effect (EPR effect), which is critical for survival, growth, evolution, invasion, and metastasis of tumor cells (Maeda et al., 2000). Owing to enhanced EPR effect and cellular internalization in tumor microenvironment, NPs could distribute and accumulate specifically into the tumor region (Maeda, 2012; Maeda et al., 2000, 2013). Therefore, novel delivery systems such as engineered nanomaterials have been developed and modified owing to the EPR effect, which allows extravasations of nanomaterials and limits the elimination of the extravasated materials (Maeda, 2012; Maeda et al., 2000, 2013). Owing to their smaller size, shorter circulation time and larger modified surface, NPs are regarded as efficient drug-delivery platform in chemotherapy. In addition, owing to the EPR effect in malignancy tissues, the concentrations of NPs are higher at the target spots. Thus, NPs have been extensively investigated for the delivery of small molecules and nucleic acids and as well as for imaging purposes. In recent years, the use of nanomedicine especially drug formulation by polymeric NPs has shown a great deal of promise to provide solutions to such problems in improving therapeutic efficacy and reducing systemic toxicity in cancer treatment (Chen et al., 2014; Long et al., 2014; Morton et al., 2014; Zhao et al., 2014). In these formulations, targeting antibodies and peptides are designed and attached to nanobiomaterials, which are biocompatible and biodegradable for binding to receptor expressed at target sites (Morton et al., 2014; Savla et al., 2014; Wang et al., 2014).

CD44, a cell adhesion molecule expressed on cancer tissue, is the principal cell surface receptor for extracellular matrix glycosaminoglycan hyaluronan (HA) (Chen et al., 2011). Our previous study revealed a linear correlation between CD44 expression level in invasive breast cancer (BC) and tumor grade (Chen et al., 2011; Wang et al., 2011). Interestingly, BC stem cells (cancer stem cell, CSCs) strongly express CD44, together with no or very low levels of CD24 (Wang et al., 2011; Kapucuoğlu et al., 2015). Cancer stem cells, which have a high proliferative potential, are responsible for tumor initiation, drug resistance, metastasis, and recurrence (Van Phuc et al., 2011; Ma et al., 2014; Im et al., 2015). Based on these facts, we have designed CD44-targeting disulfide crosslinked NPs (X-NP-DOX) based on HA-Lys-LA conjugates (Lys: l-lysine methyl ester, LA: lipoic acid) for active targeting delivery of doxorubicin (DOX). Our previous study demonstrated that X-NP-DOX had enhanced inhibitory effect on drug-resistant MCF-7/ADR tumor xenografts than free DOX (Zhong et al., 2015).

In this study, we further examined the mechanisms of the tumor inhibitory effect of low-dose X-NP-DOX delivery system on BC cells in both *in vitro* and *in vivo* studies.

## Materials and methods

### Cell culture

MCF-7 cells and T47D cells (human BC cell lines), and HCT-116 (human colorectal carcinoma cell line) were cultured in RPMI 1640 medium (Gibco BRL, Grand Island, NY, USA) containing 10% heat-inactivated fetal calf serum (Biological Industries, Israel), 1% penicillin/streptomycin, and l-glutamine. MCF-7 cells, T47D cells, and HCT-116 were assessed by Flow Cytometry Facility analysis for constitutive cell-surface CD44 expression (FITC-CD44, Miltenyi Biotec, Germany).

### Cytotoxicity assay of HA-Lys-LA_10_ X-NPs

HA-Lys-LA_10_ (degree of substitution of Lys-LA is 10) crosslinked NPs (X-NPs) were developed by our collaborator. The cytotoxicity assay of HA-Lys-LA_10_ X-NPs was performed as follows: MCF-7 cells expressing high level of CD44 receptors were seeded in a 96-well plate (1.5 × 10^4^ cells/well), and cultured with HA-Lys-LA_10_ X-NPs at various concentrations (50, 25, 12.5, 6.25, 3.125, and 1.563 mg/mL) for 4 h, the supernatant was then carefully aspirated and replaced by fresh medium. In brief, 48 h later, CCK8 solution (with a final concentration of 1.0 μg/mL) was added, the cell proliferation was measured using CCK8 assays kit by following the manufacturer’s instruction (Dojindo Laboratories, Japan).

### Confocal microscopy measurements and cellular uptake assay

Cellular uptake and intracellular drug-release behaviors of DOX-loaded HA-Lys-LA X-NPs (X-NP-DOX) were studied in MCF-7 cells using confocal laser scanning microscopy (CLSM). The cells were cultured on microscope slides placed in a 24-well plate (5 × 10^4^ cells/well) using RPMI 1640 media containing 10% fetal bovine serum, 1% l-glutamine, antibiotic penicillin (100 IU/mL), and streptomycin (100 μg/mL). After 24 h, DOX-loaded HA-Lys-LA_10_ X-NPs (X-NP-DOX) or free DOX in 100 μL of phosphate-buffered saline (PBS) was added to each well (DOX dosage, 5.0 μg/mL). After 2 or 8 h of incubation, the culture medium was removed and the cells on microscope plates were washed three times with PBS. The cells were then fixed with 4% paraformaldehyde solution for 20 min and washed with PBS containing 0.1% triton X-100 for three times. The cytoskeleton was stained with fluorescein isothiocyanate-labeled phalloidin (phalloidin–FITC, green) for 1 h and washed three times with PBS. The cell nuclei were stained with 4′,6-diamidino-2-phenylindole (DAPI, blue) for 20 min and washed with PBS. The fluorescence images were obtained using confocal microscope (TCS SP2). The inhibition experiments were performed by pretreating MCF-7 cells with free HA (5 mg/mL) for 4 h prior to incubating with X-NP-DOX.

Furthermore, T47D cells (low CD44) and HCT-116 cells (high CD44) were used to investigate the relationship between CD44 expression and cellular uptake and release behaviors of X-NP-DOX. Briefly, the cells were cultured in a 24-well plate (5 × 104 cells/well) using RPMI 1640 media containing 10% fetal bovine serum, 1% l-glutamine, antibiotics penicillin and streptomycin. After 24 h, X-NP-DOX or free DOX in 100 μL of PBS was added to each well (DOX dosage, 5.0 μg/mL). After 4 h of incubation, the culture medium was removed. The cells were then fixed with 4% paraformaldehyde solution for 20 min and washed with PBS containing 0.1% triton X-100. The cell nuclei were stained with DAPI. The fluorescence images were obtained using confocal microscope.

### Cell proliferation assays

The antitumor activity of X-NP-DOX and free DOX was also studied by CCK8 assays with the concentration of X-NP-DOX and free DOX at 0, 0.01, 0.1, 1, 10, or 100 μg/mL. After 4 h of incubation, the supernatant was carefully aspirated and replaced with fresh medium. After 48 h, CCK8 solution (Dojindo Laboratories, Japan) was added and the cell proliferation rate was determined by measuring the absorbance at 490 nm on a microplate reader (Multiskan MK3, Thermo Electron Corporation, USA).

### Cell migration and invasion assay

Cell migration was assessed by wound-healing assay (Yu et al., 2015). In brief, cells were seeded in 24-well plates, and scratched with a 200 μL pipette tip and treated with DOX or X-NP-DOX (dosage, 0.1 μg/mL), respectively. Wound areas and the movements of cells were observed after 24 h and photographed under a microscope (Axiovert 40 CFL, ZEISS).

The cell invasion assay was performed in Transwell chambers coated with BD Matrigel™ matrix (3 mg/mL) (BD Biosciences, Becton Dickson Labware, Franklin Lakes, NJ). In brief, 2.5 × 10^4^ cells were added to each 24-well invasion chamber treated with DOX or X-NP-DOX (dosage, 0.1 μg/mL). Cells incubated with PBS served as control. After 24 h of incubation, cells attached to the bottom of the membrane (migrating cells) were fixed with methanol and stained with crystal violet solution. The invasive cells were counted and the percent invasion from five representative fields was determined (Invasion (%) = [Mean number of cells invading through BD Matrigel matrix insert membrane] / [Mean number of cells migrating through uncoated insert membrane] × 100%). The mean value of triplicate assays for each experimental condition was expressed as percentage relative invasion (Park & Kim, 2015; Yu et al., 2015).

### Apoptosis analysis

Apoptosis was examined by Hoechst 33258 staining (Du et al., 2017). Briefly, MCF-7 cells (5 × 10^5^ cells/mL) were seeded in six-well plates and incubated with free DOX or X-NP-DOX with an equivalent concentration at 0.1 μg/mL for 4 h. Cells incubated with PBS served as control. After 24 h, cells were treated with Hoechst 33258 dye according to the manufacturer’s instructions (Beyotime, Shanghai, China). Apoptotic cells with intense blue nucleus were counted under fluorescence microscopy (Axio Imager.M1, ZEISS).

Meanwhile, apoptosis was also measured by an Annexin V-FITC apoptosis detection kit (Miltenyi Biotec, Germany). Briefly, MCF-7 cells or CD44^+^CD24^low/–^ cells were seeded in six-well plates and incubated with free DOX or X-NP-DOX with an equivalent concentration of DOX of 0.1 μg/mL for 4 h. Twenty-four hours later, cells were collected and stained with Annexin V-FITC, and detected with flow cytometry (CytomicsTM FC500, Beckman Coulter, Brea, CA, USA). All flow cytometry data were analyzed with FlowJO software (version 7.6.2, Ashland, OR, USA).

### Analysis of breast cancer stem cells (CD44^+^CD24^low/–^) subpopulation

MCF-7 cells (1 × 10^5^) were treated with DOX or X-NP-DOX at 0.1 μg/mL for 4 h, respectively. Twenty-four hours later, cells were harvested and incubated with anti-CD44-PE and anti-CD24-FITC for 45 min at 4 °C, followed by washing, resuspended in PBS, and analyzed by flow cytometer (CytomicsTM FC500, Beckman Coulter, USA). All flow cytometry data were analyzed with FlowJO software (version 7.6.2, Ashland, OR, USA).

To assess whether surface CD44 and CD24 expression correlates with BC CSC phenotype in our experimental system, we isolated CD44^+^CD24^low/–^ and CD44^+^CD24^+^ populations from MCF-7 cells line using CD44^+^- and CD24^+^-positive isolation kit, according to the manufacturer’s instructions (Miltenyi Biotec, Germany). Subsequently, CD44^+^CD24^low/–^ and CD44^+^CD24^+^ populations were seeded into 24-well plates (1 × 10^3^ cells/well) and cultured in DMEM/F12 medium containing 20 ng/mL of epidermal growth factor (Invitrogen, USA) and 10 ng/mL of basic fibroblast growth factor (Invitrogen, USA) (Shen et al., 2015). The cell sphere formation and metastasis ability of each cell population were then tested using light microscope (Axiovert 40 CFL, ZEISS) and transwell chambers culture system, respectively.

### Western blot analysis

MCF-7 cells (1 × 10^5^) were treated with DOX or X-NP-DOX (dosage, 0.1 μg/mL) for 4 h, respectively. Forty-eight hours later, cells were harvested for western blot analysis.

The total cell lysates were prepared using RIPA buffer (P0013B, Beyotime Biotechnology, China) supplemented with protease and phosphatase inhibitors (Sigma). Equal amounts of cellular proteins were resolved by electrophoresis in 10% of SDS–polyacrylamide gels for western blotting with specific antibodies. Antibodies used in this study were Notch1 (Abcam, clone: EP1238Y Cat.ab52627), P-Erk1/2 (Abcam, clone: 2D2. Cat. ab119776), and Erk1/2 (Abcam, clone: 9E10, ChIP Grade ab32). All antibodies in western blot assays were used by following the similar procedure as described previously (Xie et al., 2010). The blots were scanned by ChemiScope6300 (Clinx Science Instruments Co., Ltd., China).

GAPDH, beta-actin (Cat#: Mab1445, Multi Sciences Company, China), goat antirabbit IgG(H + L) HRP conjugated (Cat#: GAR007, Lot#:5104043), and goat antimouse IgG(H + L) HRP conjugated (Cat#: GAM007, Lot#:4103908) were all purchased from Multi Sciences Company, China.

### Quantitative real-time PCR

MCF-7 cells (1 × 10^5^) were treated with DOX or DOX-NP-X (dosage, 0.1 μg/mL) for 4 h, respectively. Twenty-four hours later, MCF-7 cells were collected for RNA isolation and quantitative real-time PCR analysis.

Total RNA was extracted from cells using Trizol reagent, and transcribed to cDNA with PrimeScript RT Master Mix kit (TaKaRa, Japan) by following the manufacturer’s instructions. Quantitative real-time PCR analysis of Notch1 gene expression was performed with SYBR Green I Master kit (TaKaRa, Japan) (CFX96 Touch™ Real-Time System, Bio-Rad, USA). All data were normalized using GAPDH as internal control. The gene expression was expressed as fold change from the GAPDH level which is calculated as 2 – ΔΔCt (Xie et al., 2010). In addition, melting curve analysis was performed to assure the specificity of PCR product in this experiment. The primer sequences used for Notch1 and GAPDH were (forward, 5′-CGTGTGGCCTCCTTCTACTG-3′; reverse, 5′-CTGATGCATGCGTCGTTGAG-3′) and (forward, 5′-AGAAGGTGGGGCTCATTTG-3′; reverse, 5′-AGGGGCCATCCACAGTCTTC-3′), respectively. All experiments were carried out in triplicates.

### Mouse orthotopic model setup and antitumor efficacy by X-NP-DOX

Six-week old female BALB/c nude mice were purchased from Charles River Laboratories (Beijing, China). Animals were housed in a special pathogen-free facility at the Soochow University Laboratory Animal Center. All animal protocols were conducted in accordance with the animal component of research protocol approved by Soochow University Laboratory Animal Center. Mice were injected subcutaneously into the first mammary fat pad with 1 × 10^6^ MCF-7 cells suspended in PBS with or without X-NP-DOX or free DOX per-treatment (Iorns et al., 2012). Four tumor-bearing mice injected with MCF-7 cells were used for *in vivo* imaging of X-NP-DOX. The remaining mice were used to investigate the antitumor effect of X-NP-DOX or free DOX (five per group). The tumor-bearing mice were treated with X-NP-DOX or free DOX at a dosage of 0.75 mg DOX equiv./kg given intravenously once every 2 days. Saline was used as vehicle control. Tumor volume was estimated by using the formula width^2^ × length ×0.52 in mm^3^. At day 29, mice were euthanized, and tumors were removed for further analysis. The paraffin-embedded tumors were processed for the standard histopathological examination (hematoxylin and eosin stain, or H&E) or terminal deoxynucleotidyl transferase-mediated dUTP nick end labeling (TUNEL) staining for evaluating apoptosis (Matsuu-Matsuyama et al., 2015).

In a parallel study, female BALB/c nude mice (6 weeks old) were randomly divided into the control and MCF-7 cells pretreated groups (seven per group). Prior to inoculation, MCF-7 cells were treated with free DOX or X-NP-DOX at the concentration of 0.1 μg/mL, respectively. In brief, 24 h later, each mouse was inoculated with 1 × 10^6^ MCF-7 cells per-treated DOX or X-NP-DOX in the firs mammary fat pad. Meanwhile, nontreated MCF-7 cells were also inoculated in a separated group of mice to serve as control. Tumor volume was estimated by using the formula (width + length) × 0.50 in mm^3^. At day 20, mice were euthanized, and the tumors were removed for further analysis.

### TUNEL staining assay

TUNEL assay was performed using the In Situ Cell Death Detection kit (Roche Diagnostics GmbH, Penzberg, Germany). Sections were deparaffinized and digested with proteinase K (20 μg/mL) for 15 min at 37 °C, followed by immersion with normal bovine serum and permeabilization solution. After endogenous peroxidase activity was blocked with hydrogen peroxide, sections were incubated with TUNEL reaction mixture for 1 h at 37 °C. After washing step, apoptotic cells were analyzed after incubation with Converter-POD and DAB for 30 and 10 min, respectively, and then with hematoxylin counterstaining. The percentage of TUNEL-positive cells was determined in five random fields (magnification, 400×).

### *In vivo* and *ex vivo* imaging studies

Mouse orthotopic tumor model was established as described in the previous section. To monitor the traces of NPs *in vivo*, fluorescent molecule DIR was loaded into X-NPs. When the size of tumors reached about 100 mm^3^, the tumor-bearing mice were injected with DIR-loaded X-NPs or free DIR via tail vein. Fluorescent scan was performed at various time points (1, 4, 8, 12, and 24 h) postinjection using the Maestro *in vivo* fluorescence imaging system (CRi Inc., Woburn, MA, USA).

Biodistribution of DOX delivered to the tumor and different organs was also examined via *ex vivo* imaging of DOX fluorescence. MCF-7 tumor-bearing mice were sacrificed 12 h after *i.v.* injection with X-NP-DOX or free DOX (10 mg DOX equiv./kg). The tissue blocks of tumors and several major organs including heart, liver, spleen, lung, and kidney were processed. Fluorescence images were acquired with the Maestro *in vivo* fluorescence imaging system (CRi Inc., Woburn, MA, USA).

### Statistical analysis

ANOVA test was used for analyzing the percentage of invasiveness and TUNEL-positive cells. The repeated measurements of ANOVA test were used to compare the difference of tumor volume (mm^3^) among Saline, free DOX, and X-NP-DOX groups. The *p* values of <.05 and <.01 were considered statistically significant and highly significant, respectively. All analyses were done using SAS 9.2 (SAS Enterprise Guide 3.0, Cary, NC, USA).

## Results

### CD44-targeting DOX NP delivery system *in vitro* or *in vivo*

**Figure 1.**
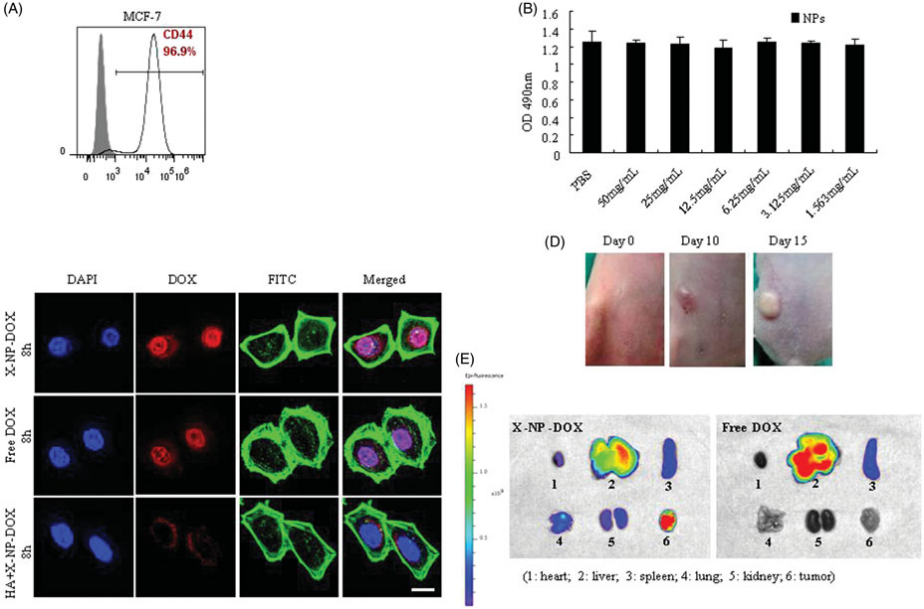
Cellular uptake, drug release *in vitro* or *in vivo*. MCF-7 cells, which express high level of CD44 receptors, were used to analyze the cytotoxicity of HA-Lys-LA10 X-NPs, the cellular uptake and intracellular drug-release behaviors of DOX-loaded HA-Lys-LA X-NPs (X-NP-DOX). Meanwhile, mouse orthotopic model was established and used to analyze the biodistribution of X-NP-DOX *in vivo*. (A) The expression of CD44 on breast cancer cell line was analyzed by flow cytometry. MCF-7 expresses high level of CD44 (96.9%). (B) The CCK8 results showed that HA-Lys-LA10 X-NPs were nontoxic toward the proliferation of MCF-7 compared with PBS. (C) CLSM analysis showed that pericellular DOX distribution in MCF-7 cells incubated with X-NP-DOX for 8 h. At 8 h, DOX fluorescence of X-NP-DOX-treated cells reached a high level, and mainly located in the nuclei as compared with that of free DOX. Free HA-pretreated MCF-7 cells displayed a remarkably low DOX fluorescence owing to the blocked binding site by free HA, confirming that X-NP-DOX was taken up by MCF-7 cells via a receptor-mediated mechanism. For each panel, the images from left to right show cell nuclei stained by DAPI (blue), DOX fluorescence in cells (red), cytoskeleton stained by phalloidin–FITC (green), and overlays of the three images. (D) Mice were injected subcutaneously into the first mammary fat pad with 1 × 106 MCF-7 cells suspended in PBS. Tumor volume was estimated by using the formula width2 × length ×0.52 in mm^3^. (E) The distribution of released DOX in tumor-bearing mice, *ex vivo* DOX fluorescence images of tumor and several major organs including heart, liver, spleen, lung, and kidney after 12-h i.v. injection of X-NP-DOX or free DOX were taken. The *ex vivo* fluorescence images showed that X-NP-DOX had strong DOX fluorescence in the tumor, which was significantly stronger than that in the major organs such as heart, liver, spleen, lung, and kidney compared with free DOX.

We tested the cytotoxicity of NPs (X-NPs) via CCK8 assays in MCF-7 cells expressing high level of CD44 (Figure 1(A)). The results showed that crosslinked NPs based on HA-Lys-LA were nontoxic toward MCF-7 cells up to 50 mg/mL compared with PBS group (Figure 1(B)), consistent with the previously reported data (Zhong et al., 2015).

Cellular uptake and intracellular drug-release behaviors of X-NP-DOX were evaluated in MCF-7 cells using CLSM. Doxorubicin fluorescence of X-NP-DOX-treated tumor cells reached the highest level at 8 h, and located mainly in the nuclei of tumor cells in comparison with that of free DOX-treated tumor cells (Figure 1(C)). In contrast, free HA-pretreated MCF-7 cells displayed a remarkable low DOX fluorescence owing to the blocked binding site by free HA (Figure 1(C)), confirming that X-NP-DOX was taken up by MCF-7 cells via a receptor-mediated mechanism. These observations are consistent with our previous observations (Zhong et al., 2015).

To confirm whether the cellular uptake and intracellular drug-release behaviors of X-NP-DOX were CD44 mediated, CD44^hi^ HCT-116 cells or CD44^lo^ T47D cells were incubated with X-NP-DOX or free DOX (Supplemental Figure S1(A)). The results showed higher pericellular DOX distribution in CD44^hi^ HCT-116 cells with X-NP-DOX compared with free DOX (Supplemental Figure S1(B)); whereas, lower pericellular DOX distribution was found in CD44^lo^ T47D cells with X-NP-DOX compared with that incubated with free DOX (Figure 1(C)). These observations suggested that X-NP-DOX was taken up by tumor cells via CD44 receptor-mediated mechanism.

To research the distribution of released DOX in tumor-bearing mice when tumor volume reached 80 mm^3^ (Figure 1(D)), *ex vivo* DOX fluorescence imaging of tumor and several major organs including heart, liver, spleen, lung, and kidney was done after 12 h *i.v.* injection of X-NP-DOX or free DOX. Mice treated with X-NP-DOX showed the strongest DOX fluorescence in the tumor compared with other major organs examined (Figure 1(E)); whereas, only weak fluorescence was seen in the tumor site in comparison with that in the liver after free DOX injection (Figure 1(E)). These results signify that X-NP-DOX can effectively target orthotopic xenografts of MCF-7 and release drug in the tumor *in vivo*.

### Low-dose X-NP-DOX suppressed migration and invasion, induced cell apoptosis and antitumor activity

**Figure 2.**
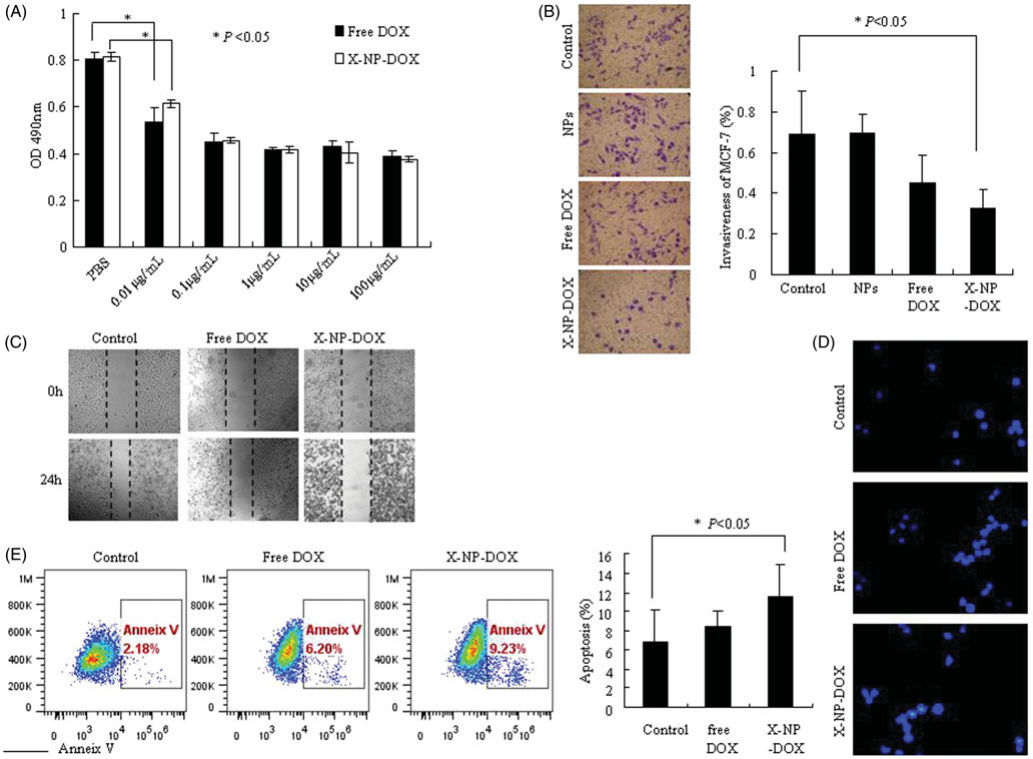
Low-dose X-NP-DOX inhibits cell growth, migration, invasion, and induces cell apoptosis *in vitro*. The CCK8 assay, wound-healing assay, and Transwell chambers assay were used to analyze cell growth, migration, and cell invasion; cell apoptosis was measured by Hoechst 33258 staining or Annexin V staining. The CCK8 assay, wound-healing assay, transwell chambers assay, and apoptosis staining was performed as described in **Materials and Methods** section. (A) The antitumor activity of X-NP-DOX in MCF-7 by CCK8 assays. Under the treatment of X-NP-DOX or free DOX, the proliferation of MCF-7 cells was markedly inhibited compared with the PBS group (*p* < .05). (B) The transwell system assay illustrated that the invasiveness of MCF-7 was inhibited by low-dose X-NP-DOX (magnification, 400×). The transwell system assay showed that the percentage of MCF-7 invasiveness treated with X-NP-DOX was significantly lower than that of PBS-treated cells (*p* < .05). (C) The wound-healing assay showed that the number of migrating cells treated with low-dose X-NP-DOX was significantly less compared to free DOX and PBS groups. (D, E) Hoechst 33258 staining (D) and Annexin V-FITC staining (E) results showed that early apoptotic cells were observed in low-dose X-NP-DOX-treated group.

We further analyzed the antitumor activity of X-NP-DOX in MCF-7 by CCK8 assays as described in **Materials and Methods** section. Under the treatment of X-NP-DOX or free DOX, the proliferation of MCF-7 cells was markedly inhibited compared with the controls at the doses of 0.01–100 μg/mL in a dose-dependent manner (Figure 2(A)). It is important to note that the proliferation of MCF-7 was inhibited by free DOX or X-NP-DOX at the lowest dose level (0.01 μg/mL) compared with control group (Figure 2(A)).

In addition to increased growth rate, invasiveness and metastasis are aggressive characteristics of malignant tumors. We thus investigated the effect of low-dose X-NP-DOX delivery system on MCF-7 cell invasion and metastasis using wound-healing assay and transwell system assay.

The transwell system assay showed that low-dose X-NP-DOX significantly suppressed the invasiveness of MCF-7 cells (Figure 2(B)). As shown in Figure 2(B), the invasiveness of MCF-7 treated with PBS, NPs, free DOX, or X-NP-DOX was 0.69 ± 0.21, 0.67 ± 0.09, 0.45 ± 0.14, and 0.32 ± 0.09%, respectively. The invasiveness of MCF-7 treated with X-NP-DOX was significantly lower than that of PBS group (*p* < .05). No difference was observed between the free DOX and the PBS group as well as NPs and PBS group (*p* > .05). In addition, the number of migrating cells in the X-NP-DOX-treated groups was found to be significantly less compared to that in free DOX- and PBS-treated cells in wound-healing assay (Figure 2(C)). These data clearly illustrated that low-dose X-NP-DOX presents remarkable antitumor effect.

Doxorubicin, as one of the most commonly used chemotherapeutic drug, inhibits DNA replication, ultimately induces cell apoptosis (Tacar et al., 2013). We therefore examined whether low-dose X-NP-DOX also enhances cell apoptosis. Hoechst 33258 staining was performed to determine the apoptosis in MCF-7 cells treated with X-NP-DOX or free DOX at 0.1 μg/mL. Untreated cells were primarily Hoechst 33258 negative, indicating that they were viable and not undergoing apoptosis. We observed increased early apoptotic cells as well as the late apoptotic cells in X-NP-DOX group 24 h post-treatment. X-NP-DOX treatment induced apoptosis, showing intense blue in the nucleus (Figure 2(D)). In contrast, weak luminescence was observed from MCF-7 cells treated with free DOX (Figure 2(D)). Furthermore, Annexin V-FITC staining confirmed that X-NP-DOX treatment produced notably higher apoptotic cells (11.49% ± 3.49) compared to control cells (6.79% ± 3.32) or DOX (8.47% ± 1.53), respectively (*p* < .05), supporting the idea that low-dose X-NP-DOX could induce enhanced apoptosis in MCF-7 cells (Figure 2(E)). Together, these results proclaimed that this NP drug-delivery system X-NP-DOX has improved antitumor effect at low dose *in vitro*.

### Low-dose X-NP-DOX inhibits CSCs of MCF-7 breast cancer cells

Cancer stem cells, which have a high proliferative potential, are responsible for tumor initiation, drug resistance, metastasis, and recurrence. Cancer stem cells of BC strongly express CD44, together with no or very low levels of CD24 (Supplemental Figure S2(A)). To identify whether surface CD44 and CD24 expression correlates with CSC phenotype, we collected CD44^+^CD24^low/–^ and CD44^+^CD24^+^ populations, and tested each population in the cell sphere formation assay. CD44^+^CD24^low/–^ MCF-7 cells grew as spheroids and cell colonies under our experimental conditions, whereas CD44^+^CD24^+^ MCF-7 cells did not (Supplemental Figure S2(B)). The transwell system assay showed that CD44^+^CD24^low/–^ MCF-7 cells were significantly higher in metastasis than that of CD44^+^CD24^+^ MCF-7 cells (Supplemental Figure S2(C)).

We then examined whether low-dose X-NP-DOX also cause enhanced CSC apoptosis. Annexin V-FITC staining revealed that X-NP-DOX treatment produced notably higher apoptotic cells (9.63% ± 1.15) compared to control cells (4.59% ± 0.82) or DOX (6.63% ± 0.91), respectively (*p* < .05), supporting the idea that low-dose X-NP-DOX could induce enhanced apoptosis in CSCs (Supplemental Figure S2(D)).

**Figure 3.**
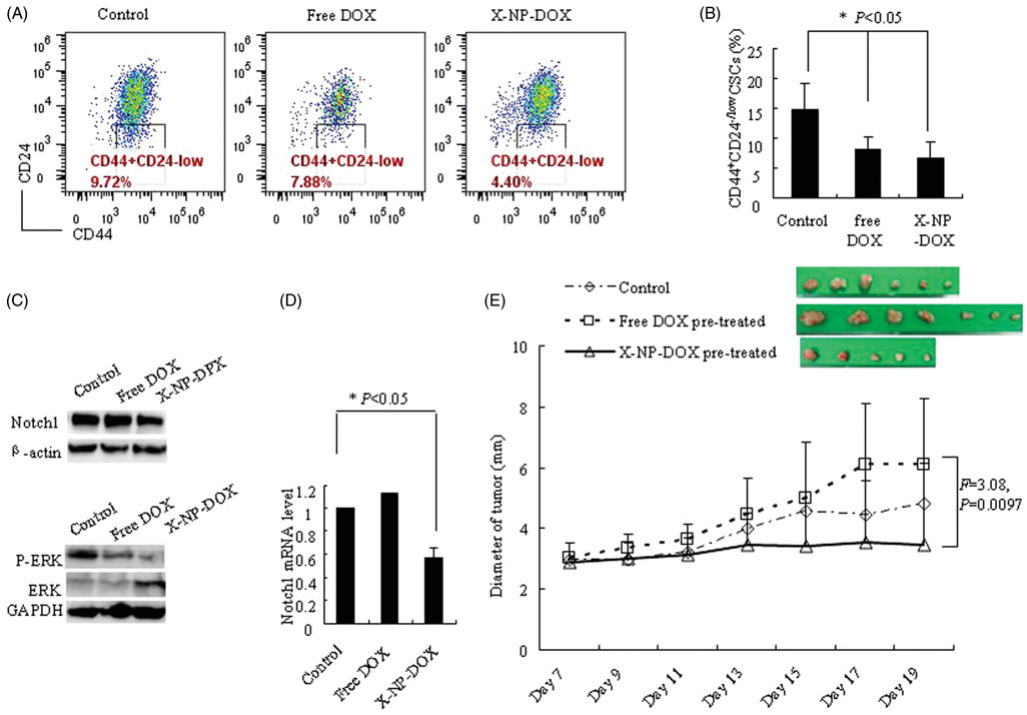
Low-dose X-NP-DOX inhibits CSCs of MCF-7 breast cancer cells. The CSC percentages assay, western blotting analysis, quantitative real-time PCR analysis, and nude mouse orthotopic breast cancer model were to further investigate the potential mechanisms of low-dose X-NP-DOX CSC inhibition. (A) The flow cytometry analysis of CSC population in MCF-7 cells with or without free DOX or X-NP-DOX pretreatment. (B) The percentages of CSCs with low-dose X-NP-DOX or free DOX were significantly lower than that of PBS-treated cells (*p* < .05). (C) Western blotting analysis revealed that low-dose X-NP-DOX strongly inhibited the expression of Notch1 and phosphor-Erk1/2 compared with free DOX or control. (D) Quantitative real-time PCR analysis showed that Notch1 gene expression was suppressed by low-dose X-NP-DOX. E: In brief, 100% mice were induced tumor injected by MCF-7 cells pretreated by DOX (7/7), whereas the percentages of detected tumor-bearing mice in control or X-NP-DOX-pretreated group were 85.7% (6/7) and 71.4% (5/7), respectively. The results showed that tumor growth was inhibited by X-NP-DOX pretreatment, whereas continuous tumor growth was observed in mice treated with free DOX (*F* = 3.08, *p* = .0097).

We further investigated the percentage of CSCs, the CD44^+^CD24^low/–^ cells, in MCF-7 cells treated with free DOX or X-NP-DOX (Figure 3(A)). The results showed that the CSC population treated with low-dose X-NP-DOX was the lowest (6.70% ± 2.65), with 8.18% ± 1.98 and 14.88% ± 4.23 in the DOX and control, respectively (*p* < .05, Figure 3(B)). Meanwhile, western blotting analysis and real-time PCR further revealed that low-dose X-NP-DOX strongly inhibited the expression of Notch1 and phosphor-Erk1/2 compared with free DOX or control group (Figure 3(C,D)). These observations point out that low-dose X-NP-DOX but not free DOX reduced the percentage of CSCs by inhibiting Notch1 and phosphor-Erk1/2 expression.

**Figure 4.**
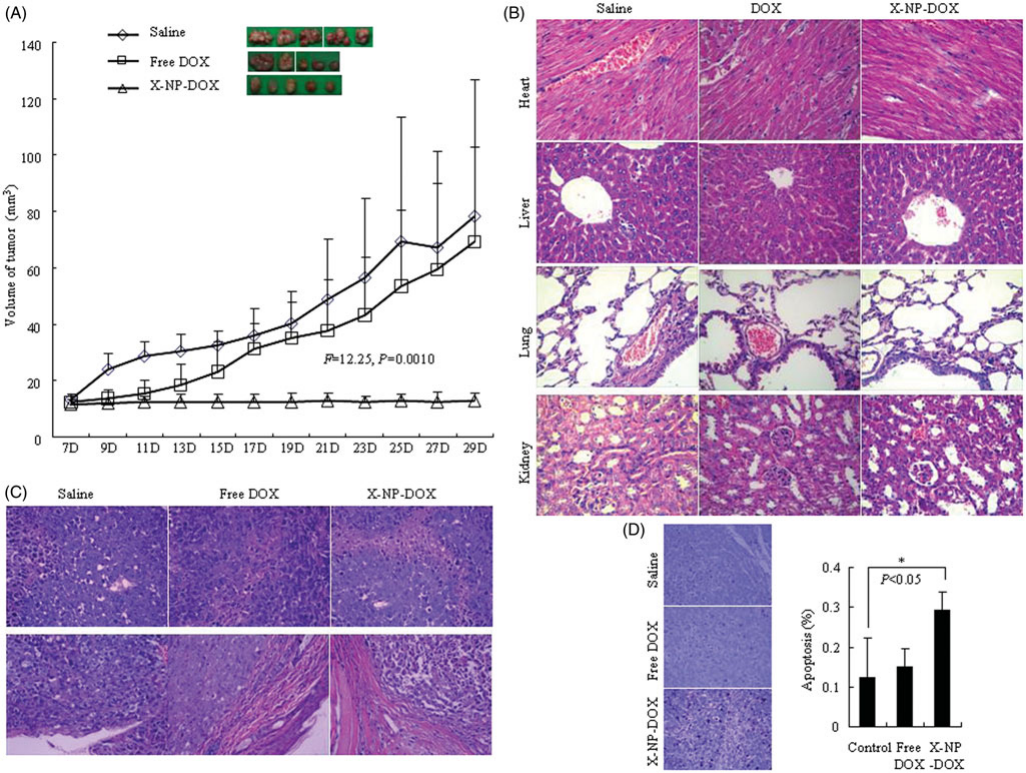
*In vivo* antitumor performance of low-dose X-NP-DOX in MCF-7 mouse orthotopic tumor-bearing model. The nude mouse orthotopic model was established to investigate the effect of low-dose X-NP-DOX on tumor growth. Tissues of orthotopic tumors, liver, lung, heart, and kidney were collected at day 29 and analyzed by H&E or TUNEL staining (magnification, 400×). (A) The results showed that tumor growth was effectively inhibited by X-NP-DOX, whereas continuous tumor growth was observed in mice treated with free DOX (*F* = 12.25, *p* = .0010). (B,C) The histological analysis using H&E staining revealed that X-NP-DOX caused widespread necrosis in the tumor tissue with no damage to the liver, kidney, spleen, and heart, and the malignant tumor cells obviously infiltrated and invaded the surrounding tissue in free DOX treatment or saline group. (D) TUNEL-positive staining of tumors in mice treated with X-NP-DOX was significant higher compared with tumors from mice treated with free DOX (*p* < .05).

To further investigate the tumorigenicity of MCF-7 treatment by X-NP-DOX or DOX, we performed *in vivo* study using orthotopic BC nude mouse model. The MCF-7 cells were pretreated with free low-dose DOX or X-NP-DOX at 0.1 μg/mL before injecting into nude mice. In brief, 100% (7 out of 7) mice developed tumor after injection with DOX-pretreated MCF-7 cells, whereas the percentages of tumor growth in control or X-NP-DOX-pretreated group were 85.7% (6/7) and 71.4% (5/7), respectively (Figure 3(E)). Furthermore, the diameter of tumor on nude mice injected with DOX-pretreated MCF-7 cells increased faster than that pretreated with X-NP-DOX (*F* = 3.08, *p* = .0097, Figure 3(E)). The results showed that tumorigenicity and tumor growth was effectively suppressed by X-NP-DOX pretreatment, whereas continuous tumor growth was observed for mice with free DOX pretreatment (Figure 4(E)).

### *In vivo* therapeutic efficacy of X-NP-DOX

In this study, we established another orthotopic BC mouse model to investigate the effect of low-dose X-NP-DOX on tumor growth. When tumors reached 12 mm^3^ in volume, mice were treated with X-NP-DOX or free DOX at low dose (0.75 mg DOX equiv./kg) or saline (control) once every 2 days. The results showed that tumor growth was effectively suppressed by X-NP-DOX, whereas continuous tumor growth was observed for mice treated with free DOX (*F* = 12.25, *p* = .0010, Figure 4(A)), likely owing to the high targetability, efficient tumor accumulation, as well as rapid drug release from X-NP-DOX. Histological examination of H&E-stained slides revealed that X-NP-DOX caused widespread necrosis in the tumor tissue with no damage to the liver, kidney, spleen, and heart (Figure 4(B,C)). In contrast, little tumor necrosis was observed in mice treated with free DOX. More importantly, there was an apparently enclosing fibrous capsule in X-NP-DOX treatment which pushed along a broad front into adjacent normal structures (Figure 4(C)). However, the malignant tumor cells obviously infiltrated and invaded the surrounding tissue in free DOX treatment or saline group (Figure 4(C)). These observations suggested that low-dose X-NP-DOX exhibits enhanced therapeutic efficacy toward CD44-positive breast tumor xenografts in mice. As DOX kills tumor cells mainly through apoptosis, we further examined this phenomenon in tumor samples by TUNEL staining. Compared with tumors from free DOX-treated mice, tumors in X-NP-DOX-treated mice had significantly higher positive TUNEL staining, confirming that X-NP-DOX induced apoptosis *in vivo* (Figure 4(D)). The positive number of apoptotic cells in X-NP-DOX group was 0.29 ± 0.10%, which was significantly higher than that of the saline group (0.12 ± 0.05%) or free DOX group (0.15 ± 0.05%) (Figure 4(D)) (*p* < .05). Together, these *in vivo* study results supported the *in vitro* observations that low-dose X-NP-DOX induces apoptotic tumor cell death.

## Discussion

Breast cancer is the prevailing cancer of women in industrialized countries, and the incidence rate in China is increasing sharply since 1993 (Redig & McAllister, 2013; Ferlay et al., 2015). Numerous therapeutic modalities are available for the treatment of breast cancer including surgery, radiotherapy, endocrine therapy, targeted therapy, and chemotherapy (Dimitrakopoulos et al., 2015; Manguso et al., 2015; Nounou et al., 2015). Although targeted therapy and endocrine therapy have improved the survival of human epidermal growth factor receptor2 (HER-2)-positive and hormone receptor-positive patients (De Laurentiis et al., 2005; Advani et al., 2015; Grassadonia et al., 2015; Manguso et al., 2015; Moya-Horno & Cortes, 2015), chemotherapy is the only realistic therapeutic approach for BC patients having aggressive tumors with negative ER or PR status. In addition, patients with hormone-sensitive tumors and HER-2 overexpression, infiltrated lymph nodes and a high proliferation rate, are also potential candidates for chemotherapy. The majority of BC incidence almost is diagnosed at 65 years or older in the Western world (Dimitrakopoulos et al., 2015). In China, the risk of BC in women aged between 40 and 60 years (Wang et al., 2011). Hence, contraindications, comorbidities, and potential toxicities should be coevaluated when selecting chemotherapy in older patients.

Doxorubicin, as a broad-spectrum antibiotic, is one of the most commonly used chemotherapeutic drugs against breast cancer, esophageal carcinomas, osteosarcoma, and lymphomas (Tacar et al., 2013). Doxorubicin has long been proven to display high antitumor efficacy, improve the quality of life and survival of patients. However, its use has been increasingly limited owing to the less amount of drug available in breast tissue even if administered at high doses (Tacar et al., 2013). Much more, DOX acts on the normal cells causing severe side effects, such as myocardial toxicity and bone marrow depression (Zhu et al., 2008; Cabeza et al., 2015). When the dosage of DOX decreases to relieve side effects, the therapeutic effect also decreases even induces drug resistance (Pasquier et al., 2010; Tacar et al., 2013; Chen et al., 2014; Shen et al., 2015). Thus, the administration of adjuvant chemotherapeutic regimens seems more difficult to maintain the balance between antitumor efficacy and dose reduction.

Nanoparticles which had huge-modified surface and shorter circulation time are regarded as novel drug-delivery system. Much more, NPs could distribute and accumulate specifically into the tumor region through by EPR effect. In this study, we showed that the uptake rate of X-NP-DOX crosslinked HA into MCF-7 cells was similar to free DOX *in vitro*. Furthermore, we demonstrated that X-NP-DOX crosslinked HA, which could bind to CD44 on the surface of CD44^hi^ HCT-116 cells or MCF-7 cells, treatment resulted in increased DOX endocytosis compared with free DOX; whereas, lower pericellular DOX distribution was seen in CD44^lo^ T47D cells with X-NP-DOX than that incubated with free DOX, suggesting that X-NP-DOX was taken up by tumor cells via CD44 receptor-mediated mechanism. When examined *in vitro*, low-dose X-NP-DOX significantly suppressed the migration and invasion of MCF-7 cells compared with free DOX. The Hoechst 33258 staining and Annexin V-FITC Flow Cytometry Analysis also showed an increase in the number of apoptotic cells treated with X-NP-DOX at low dose. More importantly, our investigation showed that the percentage of CD44^+^CD24^low/–^ CSCs significantly decreased by following X-NP-DOX treatment compared with that treated with free DOX. Notch1 is one of the Notch receptors, which plays a key role in various cell-fate decisions throughout embryonic development to adult homeostasis (Suman et al., 2013; Zhong et al., 2016). Recent studies highlighted that Notch1 and Ras/MAPK pathways are associated with development, progression, CSCs maintenance, and chemotherapy resistant of BC (Dontu et al., 2004; Zhong et al., 2016).

In BC patients, recurrence and metastasis is risk factor which leaded to poor prognosis and increased mortality. The studies have revealed that the recurrence and metastasis of human cancer and treatment failure may be partially owing to the intrinsic properties of CSCs (Wright et al., 2008). BC cells with CD44^+^CD24^low/–^, carrying the capacity of self-renewal, proliferative potential, and ability to produce differentiated progeny, are regarded as CSCs of BC (Wright et al., 2008; Ma et al., 2014; Chang et al., 2016). In tissue microenvironment, permeability and retention of EPR effect may be important to CSC to maintain survival, growth, and evolution.

In our study, gene expression and western blotting results further demonstrated that X-NP-DOX treatment inhibited both Notch1 and phospho-Erk1/2 (active MAPK). In addition, *in vivo* experiment also confirmed that tumorigenicity and tumor growth of MCF-7 cells were effectively suppressed by X-NP-DOX pretreatment. In addition, we found that the tumorigenicity of MCF-7 cells in nude mice is higher when pretreated with free DOX (7/7) than control (6/7) or X-NP-DOX (5/7). This is in accordance with the fact that patients administrated with reduced dose of DOX would be at a high risk of recurrence in clinical owing to drug resistance (Dimitrakopoulos et al., 2015). Elevated level of Notch 1 in the free DOX-pretreated group, compared with control or X-NP-DOX pretreated indicated that low dose free DOX had the potential to induce CSCs. These observations indicated that the X-NP-DOX drug-delivery system had improved antitumor effect at low dose by inhibiting invasion and metastasis, inducing apoptosis and targeting the cancer stem-like cells, decreasing the CD44^+^CD24^low/–^ CSC population *in vitro*.

We also confirmed that X-NP-DOX could effectively target, distribute, and accumulate specifically into the tumor region in MCF-7 orthotopic mouse model compared with free DOX. For this reason, further investigation *in vivo* demonstrated that the volume of orthotopic tumor was significantly smaller in the low-dose X-NP-DOX group, which could be owing to marked induction of apoptosis as shown by the result of TUNEL staining. These findings confirmed that X-NP-DOX delivery system could aim its effects directly and selectively at the tumor via inducing apoptosis or suppressing tumorigenicity *in vivo* even at low dose. The possible explanation for that X-NP-DOX has higher efficiency and selectivity in drug delivery to the tumor compared with free DOX owing to the modified surface, smaller size, and the EPR effect in malignancy tissues.

## Conclusion

In summary, X-NP-DOX as novel drug-delivery system could effectively target at BC cells via binding to CD44 molecular, and suppress cancer cells growth, inhibit migration and invasion, induce apoptosis, and reduce population of CSCs even at low dose owing to increased DOX distribution in cellular through endocytosis. These observations also illustrated that the X-NP-DOX NP delivery system may offer therapeutic opportunity for BC, at least in part, by targeting the cancer stem-like cells without serious side effects or drug resistance, and indicated that low-dose chemotherapy would be a promising treatment strategy that may be improved by NP delivery system.

## Supplementary Material

SUPPLEMENTAL

SUPPLEMENTAL
