# Supplementary figures and images for: Enhanced chemotherapeutic efficacy of the low-dose doxorubicin in breast cancer via nanoparticle delivery system crosslinked hyaluronic acid
Source: Drug Deliv. 2019 Jan 28;26(1):12–22. doi: 10.1080/10717544.2018.1507057 (PMC6352940; doi:10.1080/10717544.2018.1507057)

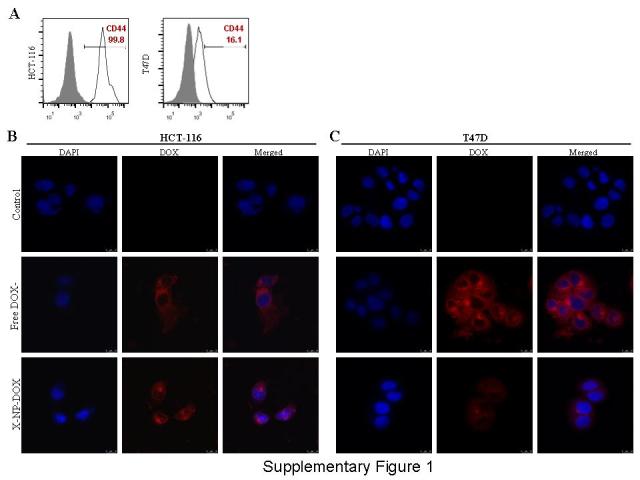

Supplement: SUPPLEMENTAL [file IDRD_A_1507057_SM0991.jpg]

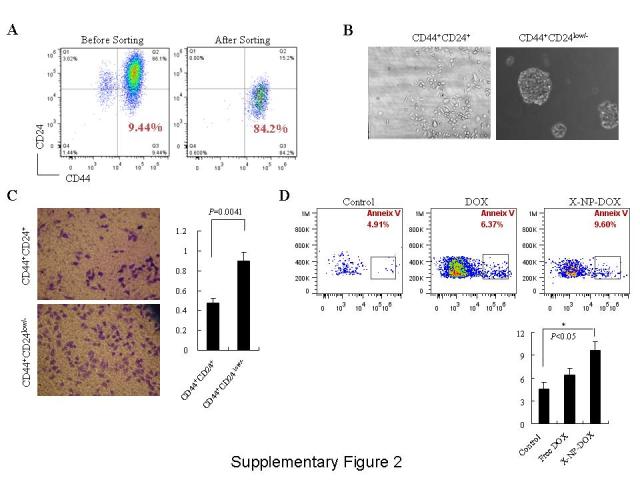

Supplement: SUPPLEMENTAL [file IDRD_A_1507057_SM0992.jpg]
